# Serum IL-27 and GDF15 levels in second trimester are associated with adverse pregnancy outcomes

**DOI:** 10.1093/jmcb/mjae053

**Published:** 2024-12-23

**Authors:** Xue Li, Luping Liu, Li Jiang, Peihong Chen, Hua Jin, Enhao Li, Jiarong Dai, Jufen Yi, Xuemei Yu, Shan Zhang

**Affiliations:** Department of Endocrinology and Metabolism, Diabetes Ward, Fengxian District Central Hospital, Shanghai 201406, China; School of Medicine, Anhui University of Science & Technology, Huainan 232001, China; Department of Endocrinology and Metabolism, Diabetes Ward, Fengxian District Central Hospital, Shanghai 201406, China; School of Medicine, Anhui University of Science & Technology, Huainan 232001, China; Department of Endocrinology and Metabolism, Diabetes Ward, Fengxian District Central Hospital, Shanghai 201406, China; School of Medicine, Anhui University of Science & Technology, Huainan 232001, China; Department of Endocrinology and Metabolism, Diabetes Ward, Fengxian District Central Hospital, Shanghai 201406, China; Department of Endocrinology and Metabolism, Diabetes Ward, Fengxian District Central Hospital, Shanghai 201406, China; Department of Endocrinology and Metabolism, Diabetes Ward, Fengxian District Central Hospital, Shanghai 201406, China; Department of Endocrinology and Metabolism, Diabetes Ward, Fengxian District Central Hospital, Shanghai 201406, China; Department of Endocrinology and Metabolism, Diabetes Ward, Fengxian District Central Hospital, Shanghai 201406, China; Department of Endocrinology and Metabolism, Diabetes Ward, Fengxian District Central Hospital, Shanghai 201406, China; Department of Endocrinology and Metabolism, Diabetes Ward, Fengxian District Central Hospital, Shanghai 201406, China

Adverse pregnancy outcomes pose significant concern for reproductive health, encompassing conditions such as gestational diabetes mellitus (GDM), postpartum hemorrhage, caesarean section, low birth weight, small for gestational age, eclampsia, preterm delivery, and peripartum fetal distress. Effectively predicting and early intervening in adverse pregnancy outcomes are imperative for ensuring the safety of both pregnant women and fetuses.

Women diagnosed with GDM experience a higher risk of specific adverse pregnancy outcomes, i.e. an increased likelihood of undergoing a caesarean section, delivering prematurely, having a low 1-min Apgar score for the newborn, and giving birth to fetal birth weight >4000 g (Caissutti et al., 2018; Ye et al., 2022). Therefore, identifying factors that predict adverse pregnancy outcomes in the gestational population is important.

Interleukin-27 (IL-27) is a heterodimer composed of EBI3 and p28 subunits and regulates immune responses with pleiotropic effects (Yoshida and Hunter, 2015). Previous studies have shown that IL-27 levels are decreased in obese individuals and IL-27 level is inversely associated with fasting blood glucose level in type 2 diabetes. Mechanistically, IL-27 directly targets adipocytes, activating p38 MAPK–PGC-1α signaling and stimulating the production of UCP1, which is a thermogenic effector (Wang et al., 2021).

Serum growth differentiation factor 15 (GDF15) is a member of TGFβ superfamily, and its expression level increases with cell stress and various pathological conditions, which can inhibit appetite and regulate glucose and lipid metabolism (Coll et al., 2020). GDF15 is required for cold-induced thermogenesis and contributes to improved systemic metabolic health following loss of OPA1 in brown adipocytes (Jena et al., 2023).

Both GDF15 and IL-27 have been implicated in metabolism and might be correlated during adipocyte thermogenesis. However, it was the first time that we analyzed the relationship between serum GDF15 or IL-27 levels in second trimester and adverse pregnancy outcomes in GDM. We aimed to explore whether IL-27 and GDF15 can be used as potential biomarkers to predict adverse pregnancy outcomes thereby contributing to the understanding and management of complications during pregnancy.

The research was a case-control study and a total of 1787 pregnant women were initially included. Further inclusion criteria were pregnant women between 14 and 28 weeks, carrying a single fetus. Next, pregnant women with assisted reproductive technology, fetal chromosome abnormalities and open neural tube defects, pre-pregnancy diabetes or hypertension, blood, heart, brain, liver, lung, kidney, and other organ diseases, autoimmune diseases or thyroid diseases, or syphilis, smoking, drug taking, and drinking histories during pregnancy were excluded. Finally, 500 participants were analyzed: 50 individuals in GDM with adverse pregnancy outcomes, 150 individuals in non-GDM with adverse pregnancy outcomes, 150 individuals in GDM with normal pregnancy outcomes, and 150 individuals in non-GDM with normal pregnancy outcomes.

The results showed that serum IL-27 levels increased in second trimester among individuals who experienced adverse pregnancy outcomes; however, there was no significant difference in serum IL-27 levels between non-GDM and GDM (Figure 1A; Supplementary Table S1), suggesting that IL-27 was not specifically associated with GDM but with adverse pregnancy outcomes. Further analysis revealed that elevated serum IL-27 levels during second trimester were associated with increased risks of preterm delivery in GDM and perinatal fetal distress in non-GDM (Supplementary Tables S2 and S3). After adjusting for common indicators, such as age, body mass index (BMI), systolic blood pressure (SBP), fasting plasma glucose (FPG), hemoglobin A1c (HbA1c), triglycerides (TG), and estimated glomerular filtration rate (eGFR), lnIL-27 represented an independent risk factor for peripartum fetal distress in non-GDM and preterm delivery in GDM (Supplementary Tables S4 and S5). The area under the peripartum fetal distress receiver operating characteristic (ROC) curve was 0.735 in non-GDM and the area under the preterm delivery ROC curve was 0.874 in GDM (Figure 1C and D; Supplementary Tables S6 and S7), supporting that serum IL-27 level in second trimester can improve the predictive efficiency of common indicators for peripartum fetal distress in non-GDM and preterm delivery in GDM. Intrauterine inflammation is associated with fetal distress and intrauterine fetal death (Manocha et al., 2019). Fetal distress is one of the main indications for cesarean section and may induce premature birth. Previous study indicated that inflammation of the fetal membrane and uterine smooth muscle mediated by the IL-27/IFN-γ/ERK axis may be associated with preterm birth (Huang et al., 2021), which aligns with our results. In this study, although IL-27 did not show a significant correlation with white blood cells and C-reactive protein, compared with non-GDM pregnant women, GDM patients had a higher risk of premature delivery. Therefore, the association between IL-27 and adverse pregnancy outcomes may be mediated through intrauterine inflammation.

**Figure 1:**
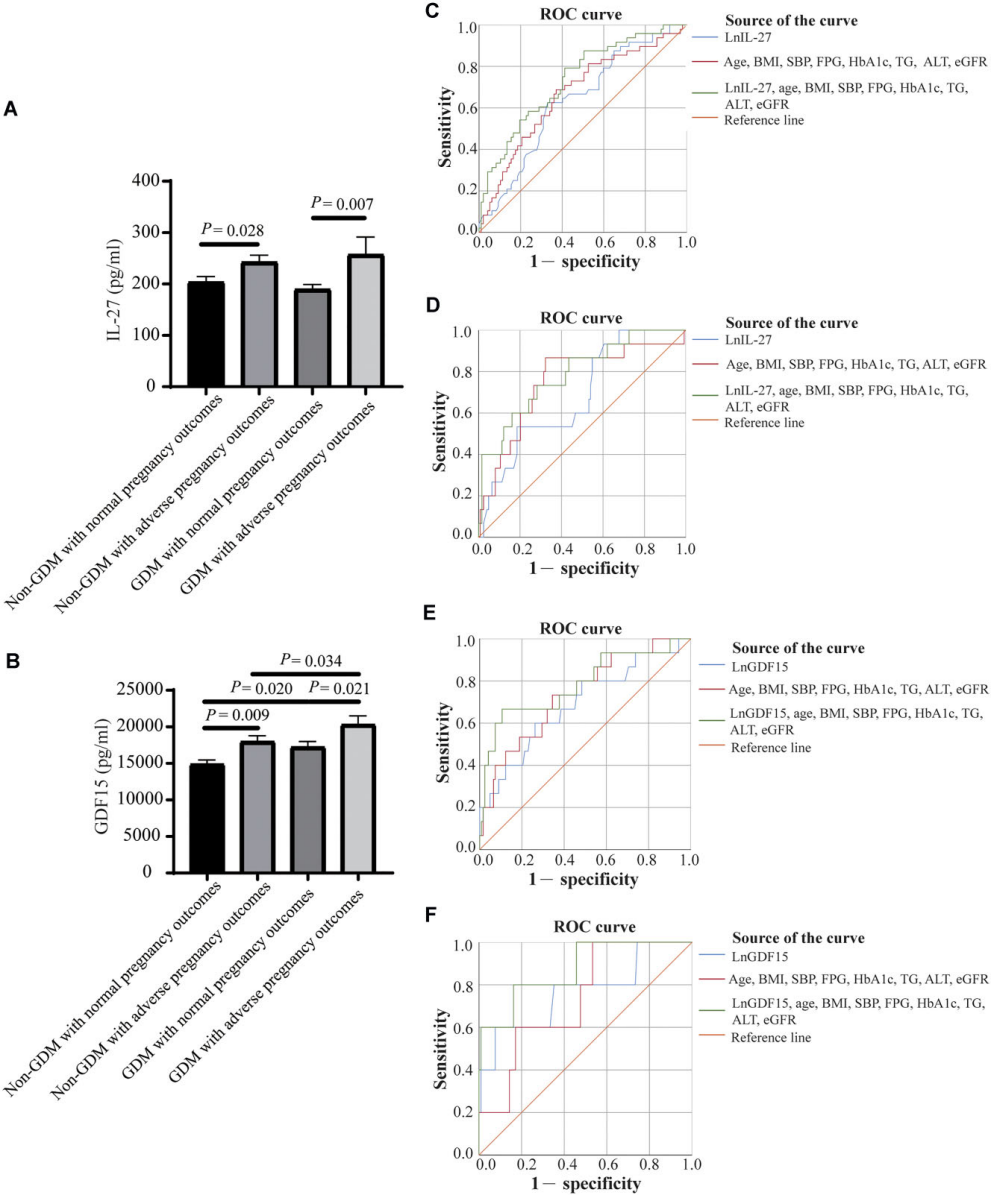
Serum IL-27 and GDF15 levels in second trimester are associated with adverse pregnancy outcomes. (**A**) Levels of IL-27 in non-GDM or GDM groups with or without adverse pregnancy outcomes. (**B**) Levels of GDF15 in non-GDM or GDM groups with or without adverse pregnancy outcomes. (**C**) ROC curves for using lnIL-27 values in the prediction of peripartum fetal distress in non-GDM group. (**D**) ROC curves for using lnIL-27 values in the prediction of preterm delivery in GDM group. (**E**) ROC curves for using lnGDF15 values in the prediction of preterm delivery in non-GDM group. (**F**) ROC curves for using lnGDF15 values in the prediction of macrosomia in GDM group.

Furthermore, our study revealed a significant correlation between serum GDF15 levels in second trimester and both GDM and adverse pregnancy outcomes. Compared with the non-GDM with normal pregnancy outcomes group, serum GDF15 levels significantly increased in all three other groups; as the number of risk factors increased, the serum GDF15 levels tended to be higher (Figure 1B; Supplementary Table S1). In addition, high serum GDF15 levels were associated with increased risks of preterm delivery in non-GDM and macrosomia in GDM (Supplementary Tables S8 and S9). After adjusting for common indicators, lnGDF15 represented an independent risk factor for preterm delivery in non-GDM and macrosomia in GDM (Supplementary Tables S10 and S11). The area under the preterm delivery ROC curve was 0.784 in non-GDM and the area under the macrosomia ROC curve was 0.787 in GDM (Figure 1E and F; Supplementary Tables S12 and S13), supporting that serum GDF15 level in second trimester can improve the predictive efficiency of common indicators for preterm delivery in non-GDM and macrosomia in GDM. GDF15 has been reported to be associated with GDM (Li et al., 2021). GDM is an important risk factor for macrosomia, which may explain why GDF15 can improve the predictive efficiency of common indicators for macrosomia in GDM. In non-GDM pregnant women, GDF15 was directly associated with preterm delivery, consistent with previous report that GDF15 level was negatively correlated with gestational age at birth in preterm infants (Almudares et al., 2023). Higher GDF15 levels were also associated with adverse respiratory outcomes in preterm infants (Almudares et al., 2023) and could restrict fetal growth and result in preterm delivery. Thus, GDF15 can not only affect pregnancy outcomes through plasma glucose but also directly induce preterm birth.

In summary, we found that elevated levels of IL-27 and GDF15 are associated with adverse pregnancy outcomes. Notably, serum IL-27 level in second trimester could improve the predictive efficiency of common indicators for peripartum fetal distress in non-GDM and preterm delivery in GDM, while serum GDF15 level in second trimester could improve the predictive efficiency of common indicators for preterm delivery in non-GDM and macrosomia in GDM. Nevertheless, achieving a comprehensive understanding of the precise mechanisms involved requires further in-depth research.

*[We thank all pregnant women participated in this study. We also thank the nurses and doctors of the Department of Endocrinology and Metabolism of Fengxian District Central Hospital for their contributions. This work was supported by grants from Shanghai Municipal Health Commission (20234Y0283 and 202040182) and Fengxian Science and Technology Commission (20191217)].*

## Supplementary Material

mjae053_Supplemental_File
