# Supplementary material for: Serum IL-27 and GDF15 levels in second trimester are associated with adverse pregnancy outcomes
Source: J Mol Cell Biol. 2024 Dec 23;16(11):mjae053. doi: 10.1093/jmcb/mjae053 (PMC12096066; doi:10.1093/jmcb/mjae053)
Supplement: mjae053_Supplemental_File [file mjae053_supplemental_file.pdf]

Materials and methods

Study participants

A retrospective analysis was made of 1787 pregnant women who were pregnant for 14-28 weeks in Fengxian District Central Hospital from May 2018 to October 2018. Among them, 241 pregnant women had adverse pregnancy outcomes, and 249 were diagnosed as gestational diabetes mellitus (GDM), 50 people had both adverse pregnancy outcomes and GDM. Taking the population diagnosed with adverse pregnancy outcomes and GDM as reference,match the age and gestational age according to the ratio of 1: 3,150 people in non-GDM with adverse pregnancy outcomes group, 150 people in GDM with normal pregnancy outcomes group and 150 people in non-GDM with normal pregnancy outcomes group were included. The adverse pregnancy outcomes included preterm delivery, pre-eclampsia, peripartum fetal distress and macrosomia. Premature delivery is that occur at less than 37 weeks' gestational age(Goldenberg et al., 2008). International guidelines agree that pre-eclampsia can be defined as new onset hypertension (systolic blood pressure sustained at ≥140 mmHg or diastolic blood pressure sustained at ≥90 mmHg, or both) with proteinuria, or end organ dysfunction after 20 weeks gestation, or both(Chappell et al., 2021). Peripartum fetal distress (Non-stress test, NST:1.Bradycardia < 100 beats/min or tachycardia > 160 beats/min for more than 30 minutes. 2.Baseline variation ≤5 beats/min for ≥80 minutes or ≥25 beats/min for > 10 minutes or sinusoidal waveform. 3.Variation deceleration lasts for ≥60 seconds or late deceleration. 4.Gestational age ≥32:more than 80 minutes, less than 2 times, and more than 15 times/minute for 15 seconds; Gestational age <32：more than 80 minutes, less than 2 times, and more than 10 times/minute for 10 seconds. Oxytocin challenge test, OCT：more than 50% of contractions are accompanied by late deceleration.) was based on the Sociaty of

Obstetricians and Gynecologists of Canada guidelines published in 2007. Macrosomia is a newborn whose weight is more than 4000 grams within 1 hour after birth(Araujo Junior et al., 2017). All participants underwent an oral glucose tolerance test (OGTT) at 24–28 weeks of gestation and blood tests for metabolic panel. The classification of GDM (fasting plasma glucose [FPG] ≥ 5.1 mmol/L or 1 h plasma glucose [1h-PG] ≥ 10.0 mmol/L, or 2 h plasma glucose [2h-PG] ≥ 8.5 mmol/L) was based on the World Health Organization guidelines published in 2013. All subjects signed an informed consent form before entering the study. Pregnant women with multiple pregnancy, assisted reproductive technology, fetal chromosome abnormalities and open neural tube defects, pre-pregnancy diabetes or hypertension, blood diseases, heart, brain, liver, lung, kidney and other organ diseases,autoimmune diseases or thyroid diseases or syphilis, smoking, drug taking and drinking history during pregnancy were excluded from this study. Informed consent was obtained from all individual participants included in the study. The Medical Ethics Committee of Shanghai Fengxian District Central Hospital approved this study, which in line with the Declaration of Helsinki.

Clinical measurements

Anthropometric parameters were collected in all subjects. The height and weight were measured by standardized method. We used the standard formula to estimate gestational age by ultrasound during the present study period, based on bi-parietal-diameter (BPD) measurements in the second trimester(Leung et al., 2008). The blood pressure was measured by a desk-top mercury sphygmomanometer. After sitting still for 10 minutes, the participants measured their blood pressure for three times and then took the average value. After an 8h overnight fast, the blood samples of all subjects were collected through the anterior elbow vein. Blood samples were

centrifuged at 1000 g for 15 minutes to obtain serum samples. Serum samples were stored at -80 ℃ before IL-27 and GDF15 detection. Alanine transaminase(ALT), aspartate aminotransferase(AST), creatinine(Cr), Uric acid(UA), total cholesterol, triglycerides (TG), low-density lipoprotein cholesterol(LDL-c), high-density lipoprotein cholesterol (HDL-c), fasting plasma glucose(FPG), 1 h plasma glucose(1hPG), 2 h plasma glucose (2hPG), were measured using an automatic biochemical analyzer (DXC 800; Beckman Coulter, Brea, CA, USA). The hemoglobin A1c(HbA1c) levels were determined by high pressure liquid chromatography (HLC-723G7; Tosoh, Tokyo, Japan). Glomerular filtration rate(eGFR) was adopted improved MDRD formula($eGFR=175*Cr^{-1.234}*(age)^{-0.179}*0.79$). All subjects were collected pregnancy outcomes.

Serum IL-27 measurement

Serum levels of IL-27 were measured by human IL-27 enzyme linked immunosorbent assay (ELISA) kit (kit#E-EL-H2338c, Elabscience Biotechnology Co.,Ltd). Serum samples were diluted 2 times before testing. The inter assay precision that CV%<10.0% and the intra assay precision CV%<10%. The kit has high sensitivity and excellent specificity. The detectable dose of human IL-27 range from 31.25-2000pg/ml. It can detect human IL-27 in the sample and has no obvious cross reaction with other analogues.

Serum GDF15 measurement

Serum levels of GDF15 were measured by human GDF15 enzyme linked immunosorbent assay (ELISA) kit (kit#sgd150, USA R & D Systems, Inc). Serum samples were diluted 100 times before testing. The inter assay precision that CV%≤6.0% and the intra assay Precision CV%≤2.8%. The kit has high sensitivity and excellent specificity. The minimum detectable dose of human

GDF15 range from 0.0-4.4pg/ml. No significant cross reaction or interference between GDF15 and analogues was observed.

Statistical analysis

All statistical analysis were performed by IBM SPSS software version 26.0(Armonk, NY, USA). Graph Pad Prism software version 8.40(La Jolla, CA, USA) were used to draw the statistical figures. Normal distribution data were presented as means±standard deviation(SD). The median (interquartile range) was used for the non normal distribution data. Independent-Sample T Test and rank sum test were used to compare the differences between two subgroups. Non-normally distributed variables were $\log_e$ converted before analysis, such as GDF15 and IL-27. One-way ANOVA and Kruskal-Wallis H(K) test were used to compare the differences among the three subgroups. The chi-square test was used for categorical variables. Binary logistic regression analysis was conducted to explore the relationship between clinical indexes and adverse pregnancy outcome. The correlations between IL-27 or GDF15 and the risk of adverse pregnancy outcome were explored by binary logistic regression analysis. Receiver operating characteristics (ROC) curves were used to calculate the predictive value of IL27 or GDF15 for adverse pregnancy outcomes. All two-tailed $p < 0.05$ were considered as significant.

Funding

This work was supported by Shanghai Municipal Health Commission (No.20234Y0283 and No.202040182) and Fengxian Science and Technology Commission (No.20191217) .

Supplementary tables and pictures

Supplementary Table 1 Characteristics of subjects in adverse pregnancy outcomes and normal pregnancy outcomes group

Variable	Non-GDM		GDM		*p*
	Normal pregnancy outcomes	Adverse pregnancy outcomes	Normal pregnancy outcomes	Adverse pregnancy outcomes	
n	150	150	150	50	
Primipara / multipara	125/25	126/24	120/30	45/5	0.420
Age（year）	28.32±3.77	28.32±4.04	29.07±3.09	29.00±3.95	0.190
Gestational week（week）	25.16±1.13	25.30±1.15	25.12±1.40	25.24±1.10	0.621
BMI（kg/m^2）	21.06±2.39	22.29±3.14	21.69±2.87	22.87±3.73	0.001
SBP（mmHg）	111.57±10.14	110.64±11.46	110.84±10.40	113.02±12.65	0.552
DBP（mmHg）	70.95±7.24	71.17±8.12	70.25±8.04	72.59±8.54	0.333
ALT（U/L）	16（11，24）	14（10，23）	14（10，22）	13（10，21）	0.502
ASL（U/L）	20（16，24）	18（16，23）	19（15，23）	18（15，21）	0.195
eGFR（ml/（min*1.73m2））	172.69±33.11	169.23±29.33	169.31±31.37	168.60±28.58	0.755
UA（umol/L）	241.55±67.22	238.57±59.84	244.21±71.54	253.11±60.27	0.607
FPG（mmol/L）	4.18±0.35	4.34±0.33	4.88±1.12ab	4.80±0.84ab	0.001
1hPG（mmol/L）	6.98±1.49	7.28±1.47	9.33±1.88ab	9.50±1.84ab	0.001
2hPG（mmol/L）	6.29±1.03	6.36±1.11	7.95±1.74ab	8.49±1.78abc	0.001
HbA1c（%）	4.79±0.34	4.87±0.32a	4.86±0.44	5.00±0.37abc	0.004
Triglycerides（mmol/L）	2.16（1.69，2.69）	2.27（1.74，2.84）	2.36(1.82,3.11)	2.28(2.03,2.96)	0.155
Total cholesterol（mmol/L）	6.29±1.07	6.16±1.04	6.21±1.04	6.12±1.00	0.682
LDL-c（mmol/L）	2.99±0.75	2.92±0.77	2.98±0.76	2.96±0.79	0.870
HDL-c（mmol/L）	1.77±0.54	1.68±0.29	1.69±0.30	1.67±0.25	0.121
IL-27（pg/ml）	203.81±114.89	242.55±141.77a	189.62±104.73b	257.20±208.75ac	0.003
GDF15（pg/ml）	14875±7157	17511±9217a	17223±9190a	20560±8682abc	0.001
Adverse pregnancy outcomes					
Preterm delivery		24 (16%)		6 (12%)	0.493
Pre-eclampsia		18 (12%)		6 (12%)	1.000
Peripartum fetal distress		80 (53.3%)		20 (40%)	0.102
Macrosomia		42 (28%)		24 (16%)	0.009

BIM： body mass index;SBP,systolic blood pressure;DBP,diastolic blood pressure;ALT,alanine aminotransferase;AST,aspartate aminotransferase; eGFR,estimated glomerular filtration rate;UA,uric acid;FPG,fasting plasma glucose;1hPG, 1h postprandial glucose; 2hPG,2h postprandial glucose;HbA1c, hemoglobin A1c;HDL-c,high-density lipoprotein cholesterol;LDL-c, low-density lipoprotein cholesterol.Data presented as the mean ± standard deviation or median (interquartile range). The 'a' represents a statistically significant difference compared with the non-GDM group with normal pregnancy outcomes. The ‘b’ represents a statistically significant difference compared with the non-GDM group with adverse pregnancy outcomes. The ‘c’ represents a statistically significant difference compared with the non-GDM group with normal pregnancy outcomes.

Supplementary Table 2 Binary logistic regression of lnIL-27 and single adverse pregnancy outcome in non-GDM

Single adverse pregnancy outcome	*OR*（*95%CI*）	*p*
Preterm delivery	1.376（0.592，3.199）	0.458
Pre-eclampsia	1.954（0.732，5.214）	0.181
Peripartum fetal distress	2.318（1.263，4.254）	0.007
Macrosomia	1.106（0.514，2.382）	0.796

Supplementary Table 3 Binary logistic regression of lnIL-27 and single adverse pregnancy outcome in GDM

Single adverse pregnancy outcome	*OR*（*95%CI*）	*p*
Preterm delivery	9.979（1.484，67.118）	0.018
Pre-eclampsia	0.370（0.084，1.626）	0.188
Peripartum fetal distress	1.402（0.490，4.009）	0.529
Macrosomia	1.979（0.752，5.211）	0.167

Supplementary Table 4 lnIL-27 was independently associated with perinatal fetal distress in non-GDM

model 1			model 2			model 3		
Variable	*OR*（*95%CI*）	*p*	Variable	*OR*（*95%CI*）	*p*	Variable	*OR*（*95%CI*）	*p*
lnIL-27	2.318（1.263，4.254）	0.007	age	0.949（0.870，1.036）	0.246	lnIL-27	3.074（1.486，6.362）	0.002
			BMI	1.077（0.945，1.228）	0.264	age	1.011（0.909，1.125）	0.836
			SBP	0.989（0.958，1.020）	0.484	BMI	1.078（0.895，1.298）	0.427
			FPG	3.699（1.32，10.120）	0.011	SBP	0.973（0.935，1.013）	0.182
			HbA1c	1.151（0.441，3.220）	0.789	FPG	7.177（1.954，26.484）	0.003
			TG	0.958（0.831，1.105）	0.556	HbA1c	0.702（0.169，2.916）	0.626
			ALT	0.998（0.983，1.013）	0.813	TG	0.900（0.567，1.431）	0.657
			eGFR	0.996（0.986，1.006）	0.434	ALT	0.999（0.982，1.016）	0.892
						eGFR	0.992（0.978，1.005）	0.238

BIM：body mass index； SBP：systolic blood pressure；ALT：alanine aminotransferase；eGFR：estimated glomerular filtration rate；FPG：fasting plasma glucose；HbA1c: hemoglobin A1c; TG：triglycerides.

Supplementary Table 5 lnIL-27 was independently associated with preterm delivery in GDM

model 1			model 2			model 3		
Variable	*OR*（*95%CI*）	*p*	Variable	*OR*（*95%CI*）	*p*	Variable	*OR*（*95%CI*）	*p*
lnIL-27	9.979（1.484，67.118）	0.018	age	0.959（0.713，1.290）	0.783	lnIL-27	20.022（1.170，342.038）	0.038
			BMI	0.879（0.583，1.327）	0.541	age	0.965（0.715，1.301）	0.813
			SBP	1.007（0.910，1.115）	0.886	BMI	0.850（0.530，1.365）	0.501
			FPG	0.505（0.086，2.948）	0.448	SBP	1.025（0.915，1.149）	0.665
			HbA1c	5.454（0.287，103.756）	0.259	FPG	0.577（0.101，3.297）	0.536

TG	1.746（0.687，4.438）	0.242	HbA1c	4.449（0.168，117.703）	0.372
ALT	0.980（0.906，1.031）	0.621	TG	1.664（0.623，4.439）	0.309
eGFR	1.000(0.967，1.034)	0.984	ALT	0.918（0.755，1.115）	0.389
			eGFR	0.991(0.950，1.034)	0.704

BIM：body mass index；SBP：systolic blood pressure；ALT：alanine aminotransferase；eGFR：estimated glomerular filtration rate；FPG：fasting plasma glucose；HbA1c: hemoglobin A1c; TG：triglycerides.

Supplementary Table 6 The ROC curves of lnIL-27 in the prediction of peripartum fetal distress in non-GDM

Variable	Area under ROC curve	*S.E*	*p*	Asymptotic 95% Confidence Interval		Sensitivity	Specificity value
				Lower Bound	Upper Boun		
lnIL-27	0.636	0.048	0.008	0.542	0.729	0.625	0.670
age,BMI,SBP,FPG,HbA1c,TG,ALT,eGFR	0.668	0.048	0.001	0.574	0.762	0.688	0.629
lnIL-27,age,BMI,SBP,FPG,HbA1c,TG,ALT, eGFR	0.735	0.043	0.001	0.65	0.821	0.792	0.588

BIM：body mass index；SBP：systolic blood pressure；ALT：alanine aminotransferase；eGFR：estimated glomerular filtration rate；FPG：fasting plasma glucose；HbA1c: hemoglobin A1c; TG：triglycerides.

Supplementary Table 7 The ROC curves of lnIL-27 in the prediction of preterm delivery in GDM

Variable	Area under ROC curve	*S.E*	*p*	Asymptotic 95% Confidence Interval		Sensitivity	Specificity value
				Lower Bound	Upper Boun		
lnIL-27	0.767	0.128	0.045	0.517	1.000	0.600	0.924
age,BMI,SBP,FPG,HbA1c,TG,ALT,eGFR	0.735	0.097	0.076	0.546	0.925	1.000	0.467
lnIL-27,age,BMI,SBP,FPG,HbA1c,TG,ALT, eGFR	0.874	0.081	0.005	0.715	1.000	0.800	0.838

BIM：body mass index；SBP：systolic blood pressure；ALT：alanine aminotransferase；eGFR：estimated glomerular filtration rate；FPG：fasting plasma glucose；HbA1c: hemoglobin A1c; TG：triglycerides.

Supplementary Table 8 Binary logistic regression of lnGDF15 and single adverse pregnancy outcome in non-GDM

Single adverse pregnancy outcome	*OR（95%CI）*	*p* 值
Preterm delivery	6.027（1.949，18.637）	0.002
Pre-eclampsia	0.771（0.278，2.138）	0.617
Peripartum fetal distress	1.216（0.728，2.029）	0.455
Macrosomia	1.184（0.621，2.257）	0.608

Supplementary Table 9 Binary logistic regression of lnGDF15 and single adverse pregnancy outcome in GDM

Single adverse pregnancy outcome	*OR*（*95%CI*）	*p* 值
Preterm delivery	8.451（0.816，87.492）	0.073
Pre-eclampsia	2.790（0.364，21.373）	0.323
Peripartum fetal distress	1.346（0.511，3.542）	0.548
Macrosomia	3.754（1.269，11.107）	0.017

Supplementary Table 10 lnGDF15 was independently associated with preterm delivery in non-GDM

model 1			model 2			model 3		
Variable	*OR*（*95%CI*）	*p*	Variable	*OR*（*95%CI*）	*p*	Variable	*OR*（*95%CI*）	*p*
lnGDF15	6.027（1.949，18.637）	0.002	age	1.094(0.918,1.305)	0.315	lnGDF15	4.169(1.147,15.158)	0.03
			BMI	1.138(0.855,1.515)	0.374	age	1.091(0.906,1.314)	0.358
			SBP	0.925(0.867,0.986)	0.018	BMI	1.172(0.863,1.590)	0.309
			FPG	1.017(0.157,6.594)	0.986	SBP	0.932(0.871,0.997)	0.039
			HbA1c	2.333(0.397,13.698)	0.348	FPG	0.590(0.078,4.471)	0.610
			TG	0.935(0.449,1.948)	0.858	HbA1c	2.431(0.336,17.592)	0.379
			ALT	0.968(0.918,1.020)	0.226	TG	0.815,0.361,1.843	0.624
			eGFR	1.011(0.992,1.030)	0.259	ALT	0.974(0.923,1.028)	0.337
						eGFR	1.016(0.994,1.038)	0.159

BIM：body mass index；SBP：systolic blood pressure；ALT：alanine aminotransferase；eGFR：estimated glomerular filtration rate；FPG：fasting plasma glucose；HbA1c: hemoglobin A1c; TG：triglycerides.

Supplementary Table 11 lnGDF15 was independently associated with macrosomia in GDM

model 1			model 2			model 3		
Variable	*OR*（*95%CI*）	*p*	Variable	*OR*（*95%CI*）	*p*	Variable	*OR*（*95%CI*）	*p*
lnGDF15	3.754（1.269，11.107）	0.017	age	1.064(0.877,1.290)	0.531	lnGDF15	5.119(1.221,21.462)	0.026
			BMI	1.134(0.917,1.403)	0.246	age	1.016(0.836,1.235)	0.874
			SBP	0.972(0.918,1.028)	0.317	BMI	1.153(0.900,1.478)	0.260
			FPG	1.302(0.754,2.245)	0.344	SBP	0.979(0.920,1.042)	0.509
			HbA1c	5.158(1.106,24.046)	0.037	FPG	1.300(0.755,2.241)	0.344
			TG	0.791(0.417,1.502)	0.474	HbA1c	4.710(0.892,24.868)	0.068
			ALT	1.021(1.000,1.042)	0.053	TG	0.753(0.372,1.526)	0.432
			eGFR	1.014(0.996,1.033)	0.137	ALT	1.018(0.997,1.040)	0.093
						eGFR	1.010(0.990,1.029)	0.329

BIM：body mass index；SBP：systolic blood pressure；ALT：alanine aminotransferase；eGFR：estimated glomerular filtration rate；FPG：fasting plasma glucose；HbA1c: hemoglobin A1c; TG：triglycerides.

Supplementary Table 12 The ROC curves of lnGDF15 in the prediction of preterm delivery in non-GDM

Variable	Area under ROC curve	*S.E*	*p*	Asymptotic 95% Confidence Interval		Sensitivity	Specificity value
				Lower Bound	Upper Boun		
lnGDF15	0.689	0.078	0.017	0.537	0.841	0.600	0.738
age,BMI,SBP,FPG,HbA1c,TG,ALT,eGFR	0.736	0.067	0.003	0.603	0.868	0.733	0.656
lnGDF15,age,BMI,SBP,FPG,HbA1c,TG,ALT, eGFR	0.784	0.072	0.001	0.642	0.926	0.667	0.893

BIM：body mass index；SBP：systolic blood pressure；ALT：alanine aminotransferase；eGFR：estimated glomerular filtration rate；FPG：fasting plasma glucose；HbA1c: hemoglobin A1c; TG：triglycerides.

Supplementary Table 13 The ROC curves of lnGDF15 macrosomia in GDM

Variable	Area under ROC curve	*S.E*	*p*	Asymptotic 95% Confidence Interval		Sensitivity	Specificity value
				Lower Bound	Upper Boun		
lnGDF15	0.684	0.066	0.020	0.554	0.814	0.533	0.815
age,BMI,SBP,FPG,HbA1c,TG,ALT,eGFR	0.752	0.071	0.001	0.631	0.892	0.867	0.677
lnGDF15,age,BMI,SBP,FPG,HbA1c,TG,ALT, eGFR	0.787	0.063	0.001	0.664	0.909	0.733	0.718

BIM：body mass index；SBP：systolic blood pressure；ALT：alanine aminotransferase；eGFR：estimated glomerular filtration rate；FPG：fasting plasma glucose；HbA1c: hemoglobin A1c; TG：triglycerides.

Reference:

1 Araujo Junior, E., Peixoto, A.B., Zamarian, A.C., et al. (2017). Macrosomia. Best Pract Res Clin Obstet Gynaecol *38*, 83-96.

2 Chappell, L.C., Cluver, C.A., Kingdom, J., et al. (2021). Pre-eclampsia. Lancet *398*, 341-354.

3 Goldenberg, R.L., Culhane, J.F., Iams, J.D., et al. (2008). Epidemiology and causes of preterm birth. Lancet *371*, 75-84.

4 Leung, T.N., Pang, M.W., Daljit, S.S., et al. (2008). Fetal biometry in ethnic Chinese: biparietal diameter, head circumference, abdominal circumference and femur length. Ultrasound Obstet Gynecol *31*, 321-327.
